# Deconstructing stereotypes: Stature, match-playing time, and performance in elite Women's World Cup soccer

**DOI:** 10.3389/fspor.2022.1067190

**Published:** 2022-12-14

**Authors:** Ciara N. Manning, Yasuki Sekiguchi, Courteney L. Benjamin, McKenna R. Spaulding, Erin E. Dierickx, Jayson M. Spaulding, Dayshia M. Davenport, Jillian R. Picard-Busky, George T. Chiampas, George P. Nassis, Douglas J. Casa

**Affiliations:** ^1^ Department of Kinesiology, Korey Stringer Institute, University of Connecticut, Storrs, CT, United States; ^2^ Sports Performance Lab, Department of Kinesiology and Sport Management, Texas Tech University, Lubbock, TX, United States; ^3^ Department of Kinesiology, Samford University, Birmingham, AL, United States; ^4^ Department of Health and Physical Education and Recreation, Dickinson College, Carlisle, PA, United States; ^5^ Department of Exercise Science and Athletic Training, Ithaca College, Ithaca, NY, United States; ^6^ School of Kinesiology, Louisiana State University, Baton Rouge, LA, United States; ^7^ Department of Exercise Science, Sacred Heart University, Fairfield, CT, United States; ^8^ Department of Emergency Medicine, Northwestern University, Chicago, IL, United States; ^9^ Chief Medical Officer, United States Soccer Federation, Chicago, IL, United States; ^10^ Physical Education Department, College of Education, United Arab Emirates University, Al Ain, UAE; ^11^ Department of Sports Science and Clinical Biomechanics, SDU Sport and Health Sciences Cluster (SHSC), University of Southern Denmark, Odense, Denmark

**Keywords:** women's soccer, height, performance, FIFA world cup, playing time

## Abstract

Recruiting companies recommend elite female soccer players be ≥165 cm (5′5″) in stature. This study investigated if stature limits match-playing time and performance in elite World Cup soccer among players, positions, and countries. We hypothesized stature would not affect match-playing time or performance. Descriptive data were collected on 552 players from 2019 FIFA Women's World Cup. Odds ratios determined likelihood of starting for players <165 cm and ≥165 cm. ANOVAs compared playing time between stature groups, among positions, and between countries. Performance factors including assists, goals, attempts, corners, shots blocked, and defending blocks were reported. Independent t-tests compared differences between players (≥165 cm, < 165 cm). Data are reported, mean difference [95% confidence interval] [MD (95%CI)] and effect sizes (ES). On average, 32.3% of players were <165 cm. Of total players, no differences existed in total minutes (*F = *0.98, *p* = 0.32)*,* matches (*F* = 0.27 *p *= 0.59), or average minutes per match (*F = *0.48, *p *= 0.49) between stature groups, regardless of position. No differences existed in playing time between players <165 cm and ≥165 cm among any positions (*p* > 0.05), or between countries (*p* > 0.05). Taller mid-fielders exhibited greater performance in goals, assists, attempts, shots blocked, and defending blocks (MD [95%CI] ES; assists, −0.44[−0.76,−0.11]0.59, *p* = 0.009; goals, −0.35[−0.69,−0.01]0.44, *p* = 0.047); attempts, 3.14[1.38, 4.90]0.80, *p* = 0.001; corners, 2.04[0.12, 3.95]0.48, *p* = 0.037; shots blocked, 0.96[0.40, 1.51]0.75, *p* = 0.001; defending blocks, 0.43[0.32,0.82]0.48, *p* = 0.035), however, actual differences were minimal. Our findings indicate stature does not inhibit playing and performing elite women's soccer, as nearly one-third of players were <165 cm.

## Introduction

The International Federation of Association Football (FIFA), the largest governing body in the sport, organizes the FIFA World Cup tournaments for qualifying teams all over the world to compete in every four years (1). Many youth athletes may aspire to play at this elite level of soccer. The number of high-school soccer players in the United States has increased 10.7% for females and 17.2% for males in the past decade (1). A small number of these high-school athletes make it to the collegiate and professional level (1, 2). Of the 390,482 female high-school soccer players reported in 2017–2018, only 27,811 (7.1%) continued playing at the National Collegiate Athletic Association (NCAA) level (2). The estimated probability of performing at the collegiate level after high-school in 2019 was 2.4% for Division I, 1.9% for Division II, and 2.8% for Division III (2). This low probability raises the question of what it takes to reach collegiate and elite level success, and what coaches and colleges look for in potential athletes.

College recruiting companies list minimal stature guidelines of ≥165 cm for female high-school athletes who aspire to play at the NCAA level (3). Provisions also include that Division I players be of the following statures: 170 cm–188 cm (5′7″–6′2″) for goalkeepers, ≥ 168 cm (5′6″+) for outside defenders, 170 cm–188 cm (5′7″–6′2″) for center defenders, and ≥165 cm (5′5″+) for outside mid/wing forwards (3). No stature is listed for center midfielders or forwards but can be implied based on requirements for other positions (3). These guidelines set the basis and understanding of what to strive for, for both athletes and coaches. Stating such guidelines and creating stereotypes for soccer players may discourage talented athletes from pursuing soccer at the elite level, strictly on the basis of their stature (4). However, it's not currently known if stature impacts match-playing time or performance in elite level soccer.

Generally, it is a common assumption that players of taller stature are more likely to succeed in elite-level soccer (5). However, in contradiction, some studies have found sub-elite soccer players to be of greater stature than their elite counterparts, suggesting that stature may not always characterize elite status or performance (6). Sports performance research has yet to fully define main attributes and actions that differentiate successful soccer players from unsuccessful soccer players (7–13). Limited research has been done assessing what constitutes match-playing time in soccer and why some players receive more playing time than others.

Many researchers have tried to determine what results in athletes being selected to play at an elite level, and what should constitute the selection process to ensure that selection is based on objective rather than subjective measures (14–17). In a recent systematic review, researchers operationalized multiple criterion variables that exist in soccer to determine their level of application in the actual playing soccer (18). Of the criterion variables, stature was indicated as having little overlap in the actual playing of soccer (18). Regardless of its low applicability in relation to match-play, stature is commonly used by recruiting companies and coaches to identify talented soccer players (3, 18).

Anatomical and biomechanical differences have been considered partially influential in soccer performance in regards to the skill of kicking and certain running motion patterns (19). Longer limbs have the ability to produce more force, torque, and can produce a greater change in momentum.(19–24) This is believed to relay to soccer performance in kicking, jumping, and running (19). The anthropometric profile of soccer players has also been found to differ between positions suggesting that physical characteristics vary by playing position (17, 25–27). Certain statures and ranges of body mass have been deemed beneficial to players of different positions in elite-level soccer (25, 28). Anthropometric factors including stature, body mass, % body fat, and sum of skinfolds tend to decrease among positions ranging from goal keepers with the highest values, followed by defenders, forwards, and midfielders having the lowest values (25). Previous research suggests that heavier and taller players had greater vertical jump and 30-m sprinting abilities required by positions of goal keepers, defenders, and forwards (25, 28). This research also reported that players of lower BMI had greater aerobic endurance required of external defenders and central and external midfielders (25, 28).

No research has been done on the duration of match-playing time in relation to one's stature. While recruiting companies list minimal stature provisions of ≥165 cm (5′5″) for aspiring female soccer players, it is not currently known whether or not stature impacts the duration of match-playing time in soccer at the elite World Cup level. Therefore, the primary aim of this study is to determine if stature is a limitation to the duration of match-playing time on an elite World Cup soccer team. We hypothesized that stature (≥165 cm or <165 cm) would have no effect in the duration of match-playing time among players. Additionally, no research has been done assessing the impact of stature on performance in elite women's World Cup soccer. Therefore, the secondary aim of this study is to determine if differences in performance exist among elite female soccer players of different stature (≥165 cm and <165 cm) among all positions in the 2019 FIFA Women's World Cup. We hypothesized that no performance differences would exist among players of different stature within all positions.

## Materials and methods

### Participants

This study utilized an observational, cross-sectional design. Data were obtained from the official website of FIFA World Cup and the records of the 2019 Women's World Cup (29). Statistics from 552 female soccer players from 24 teams who played in the 2019 FIFA World Cup games were reported on the website and manually recorded in an Excel sheet for this analysis. Variables obtained from the website included player's name, age, stature, position, minutes played, matches played, and performance factors (29). Stature was self-reported by each team. On average players were 27.14 ± 3.96 years old. Players were categorized as those ≥165 cm (5′5″) and those <165 cm (5′5″). Since the collection of data for this manuscript, the website has been updated and no longer reports individual demographics of individual players, but rather general team statistics (29). Physical analysis of player demographics can be found in the Physical Analysis of the FIFA Women's World Cup 2019 report and on the U.S. soccer website for the U.S. team (30, 31).

### Likelihood of playing

All 552 female soccer players were included to assess the amount of time spent in match-play in relation to stature. Descriptive data on matches played and minutes played for each player were analysed. The data were categorized into those below and those at or above the 165-cm stature criterion. Odds ratios were calculated to determine if differences existed in the likelihood of being a starter for those players on the team <165 cm tall and those on the team ≥165 cm tall. Categorizing players as starters or non-starters allowed for identification and comparison of players who typically receive the longest duration of match-play time with players who receive less match-play time. Researchers have used various criteria when classifying players as starters and non-starters with some researchers classifying starters as those who play for 83% of total game time and non-starters as those who play for 17% of total game time (32). Other researchers have classified starters as those who regularly participate in practices (33), or those who play > 60% of games (34). Additionally, researchers have considered starters as those playing for 45 min of total game time and non-starters as those who play less (35, 36). In our analysis, players were considered starters if they played ≥60 min when entered into a match. We believe this is an appropriate classification as this would equate to players who played a majority (roughly 67%) of the duration of a match. This was defined as a significant amount of playing time. A one-way ANOVA was used to examine if stature impacted the number of minutes played, number of matches played, average minutes played when entered into a match, average minutes played in relation to the total number of matches the team played during the entire 2019 FIFA Women's World Cup, and percent playing time between all players <165 cm and ≥165 cm (Table 1). Normality was not necessary for these analyses given the large sample size (37).

**Table 1:** Playing time and performance measures for all players ≥165 cm and <165 cm.

Playing Time Measures	≥165 cm	<165 cm	Statistics
Total minutes played	195.10 ± 185.66	179.46 ± 146.99	*F *= 0.98, *p = 0.32,* ES = 0.09
Number of matches played	2.65 ± 2.08	2.56 ± 1.66	*F = *0.27, *p = 0.59,* ES = 0.05
Average minutes played per match	52.73 ± 37.64	55.04 ± 34.45	*F = *0.48, *p = 0.49,* ES = 0.05
Average minutes played in relation to total minutes team played in	42.26 ± 36.50	45.12 ± 34.14	*F = *0.78, *p = 0.38*, ES = 0.08
Percentage of playing time	46.95% ± 40.56	50.14% ± 37.93	*F = *0.78, *p *= 0.38, ES = 0.06
**Performance Measures**			
Goals (number)	0.57 ± 1.17*	0.25 ± 0.62	*F = 16.98, p = 0.01, MD = *−*0.32, 95% CI [*−*0.58,* −*0.07]*
Assists (number)	0.32 ± 0.69	0.22 ± 0.55	*F = 4.71, p = 0.25, MD = *−*0.10, 95% CI [*−*0.26, 0.07]*
Attempts (number)	4.55 ± 5.27*	3.22 ± 3.56	*F = 9.57, p = 0.03, MD = *−*1.33, 95% CI [*−*2.52,* −*0.13]*
Corners (number)	1.79 ± 4.53	1.18 ± 3.07	*F = 6.00, P = 0.25, MD = *−*0.61, 95% CI [*−*1.64, 0.42]*
Distance covered per match (km)	9.13 ± 1.27	8.97 ± 1.84	*F = 5.32, p = 0.41, MD = *−*0.16, 95% CI [*−*0.54, 0.22]*

Data are presented as averages ± standard deviation, *F*-value, *p*-value, mean difference, and 95% confidence interval.

* Indicates statistically significant differences (*p* < 0.05) between stature groups.

### Match-playing time

For each player, average minutes played per match, average minutes played in relation to the total number of matches their team played in during the World Cup, and percentage of playing time were manually computed (29, 38). Average minutes played per match was calculated by dividing the total number of minutes an individual played by the number of matches that the individual played (9, 38, 39). This was calculated to assess how much playing time, if given the opportunity by being entered into a match, individuals received. Average minutes played in relation to the total number of matches the team played in during World Cup was calculated by dividing the total number of minutes an individual played by the number of matches that the team played in during the World Cup (9, 29, 38, 39). This was calculated to analyse playing time on the basis of team opportunity rather than on an individual level. Percentage of playing time was calculated by dividing the number of minutes an individual played by the total number of minutes their team played during the World Cup and multiplying by 100. This was calculated as another means to compare playing time between height groups.

### Positions and playing time

Players were separated by position to determine how many players in each position were <165 cm and ≥165 cm tall. Four positions were included in this analysis: forwards, defenders, midfielders, and goal keepers. Ratios were calculated to determine percentages of players in each position and each stature group. A multivariate ANOVA was used to examine differences in stature, position, and stature among positions (Table 2). This determined if stature impacted the number of minutes played, number of matches played, average minutes individuals played when entered into a match, average minutes individuals played in relation to the total number of matches their team played in during the entire 2019 FIFA Women's World Cup, and percentage of playing time between players <165 cm and ≥165 cm tall, and within each position. LSD *post hoc* comparisons were used when appropriate to determine where specific differences occurred.

**Table 2:** Playing time for defenders, forwards, and midfielders ≥165 cm and <165 cm.

Position	≥165 cm	<165 cm	Statistics
**Defenders**			
Minutes played	220.73 ± 195.08	179.78 ± 149.98	MD = −45.78, 95% CI = [−99.65, 8.08], ES = 0.25, *p = 0.10, t = *−*1.68,*
Matches played	2.73 ± 2.06	2.57 ± 1.56	MD = −0.53, 95% CI = [−1.16, 0.95], ES = 0.25, *p = 0.10,* *t = *−*1.67*
Average minutes per match	60.24 ± 37.95	56.79 ± 33.58	MD = −5.25, 95% CI = [−17.55, 7.05], ES = 0.14, *p = 0.40, t = *−*0.84*
Average minutes per total matches team played	47.49 ± 37.84	46.42 ± 34.60	MD = −2.93, 95% CI = [−14.98, 9.12], ES = 0.08, *p = 0.63, t = *−*0.48*
**Forwards**			
Minutes played	206.35 ± 156.57	188.24 ± 38.47	MD = −18.11, 95% CI = [−71.97, 35.75], ES = 0.12, *p = 0.51, t = *−*0.67*
Matches played	3.18 ± 1.86	3.04 ± 1.65	MD = −0.14, 95% CI = [−0.78, 0.50], ES = 0.08, *p = 0.66, t = *−*0.44*
Average minutes per match	55.53 ± 30.65	52.62 ± 28.21	MD = −2.90, 95% CI = [−13.57, 7.76], ES = 0.10, *p = 0.59, t = *−*0.54*
Average minutes per total matches team played	45.74 ± 31.39	44.63 ± 29.46	MD = −1.11, 95% CI = [−12.10, 9.88], ES = 0.04, *p = 0.84, t = *−*0.20*
**Midfielders**			
Minutes played	194.69 ± 186.69	179.78 ± 149.98	MD = −14.92, 95% CI = [−67.12, 37.27], ES = 0.20, *p = 0.57, t = *−*0.57*
Matches played	2.93 ± 2.02	2.57 ± 1.56	MD = −0.36, 95% CI = [−0.92, 0.19], ES = 0.09, *p = 0.20, t = *−*1.29*
Average minutes per match	51.11 ± 34.06	56.79 ± 33.58	MD = 5.68, 95% CI = [−4.87, 16.23], ES = 0.17, *p = 0.29, t = 1.06*
Average minutes per total matches team played	40.27 ± 34.72	46.42 ± 34.60	MD = 6.14, 95% CI = [−4.66, 16.95], ES = 0.18, *p = 0.26, t = 1.12*

Data are presented as averages ± standard deviation, mean difference (MD), and 95% confidence interval (95%CI), effect size (ES), *p*-value, and *t*-value.

### Performance factors

Data for players was further separated from stature, by position. Of the total 552 female soccer players, 288 players (93 players <165 cm) were included when assessing performance. Players were only included if they played an average of ≥60 min when entered into a match as this was representative of athletes who play a majority (roughly 67%) of the duration of a match (32–36). Positions were classified as forwards, midfielders, and defenders. No goal keepers were <165 cm, therefore, they were not included in the analysis. Performance was determined in terms of number of assists, number of goals, number of attempts, attempts on target, attempts off target, attempts inside the area, attempts outside the area, attempts on target inside area, attempts on target outside the area, number of corners, shots blocked, defensive blocks, and average distance covered per match played. Average distance covered per match played was a manually computed variable calculated for each player by dividing the total distance covered in kilometres during the World Cup games by the number of matches that individual played in during the World Cup. The averages of each performance factor per player were calculated to compare differences between the two stature groups. Independent t-tests were used to examine differences in each of these factors in players ≥165 cm and <165 cm of all positions (Table 1).

### Countries

Each of the 24 teams that competed in the 2019 FIFA Women's World Cup were included in this analysis to determine the percentage of players from each team that were <165 cm and ≥165 cm tall. Each team consisted of 23 players. Ratios were calculated to determine the percentage of starters on each team that were <165 cm. A one-way ANOVA was used to determine differences in playing time between countries.

All statistical analyses were performed using SPSS Statistics Version 26 (IBM Corp., Armonk, NY). Statistical significance was set at *p *< 0.05, *a priori*. *Hedge's g* effect size was determined. Effect size was identified as small (0.05–0.49), moderate (0.50–0.79), or large (≥0.80) (40).

## Results

**Figure 1:**
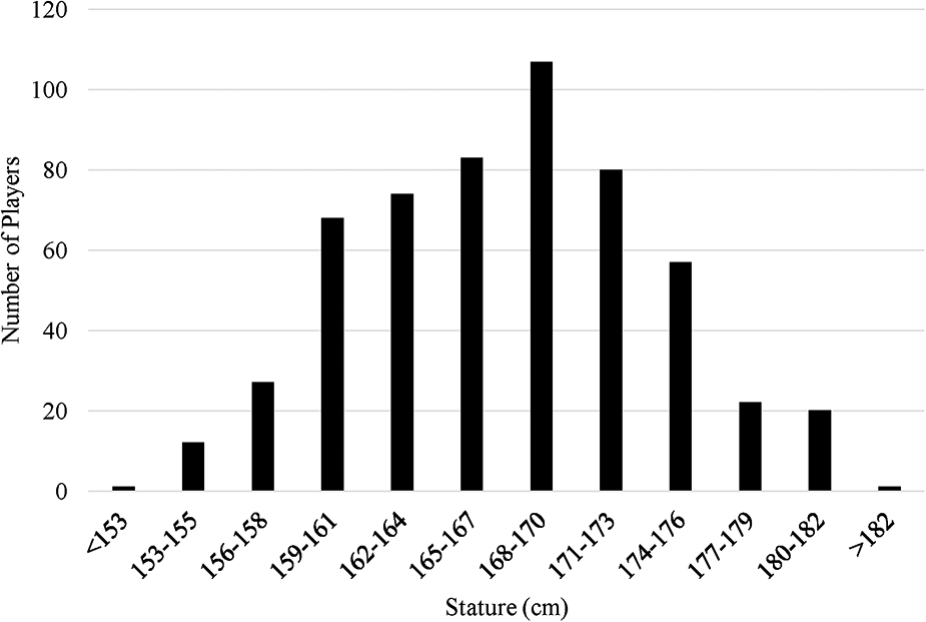
Overall breakdown of the statures of 552 players representing 24 countries in the 2019 FIFA Women's world Cup games.

Data are reported as mean difference [95% confidence interval] [MD (95%CI)] and effect sizes (ES), or in some cases (*F-*value, *p-*value) and effect sizes (ES). In total, there were 178 players <165 cm and 374 players ≥165 cm. Of the total 552 female soccer players, 288 players were starters. Of the starters, 93 players (32.29%) were <165 cm (Figure 1).

### Playing time

For players on the team rosters, the odds of starting if they were <165 cm (52.20% of all players <165 cm) were equivalent to the odds of starting if they were ≥165 cm (52.10% of all players ≥165 cm); odds ratio [95% confidence interval] (OR [95%CI], 1.0 [0.70, 1.42]). No statistically significant differences were found in playing time between those <165 cm and those ≥165 cm (Table 1).

### Position

**Figure 2:**
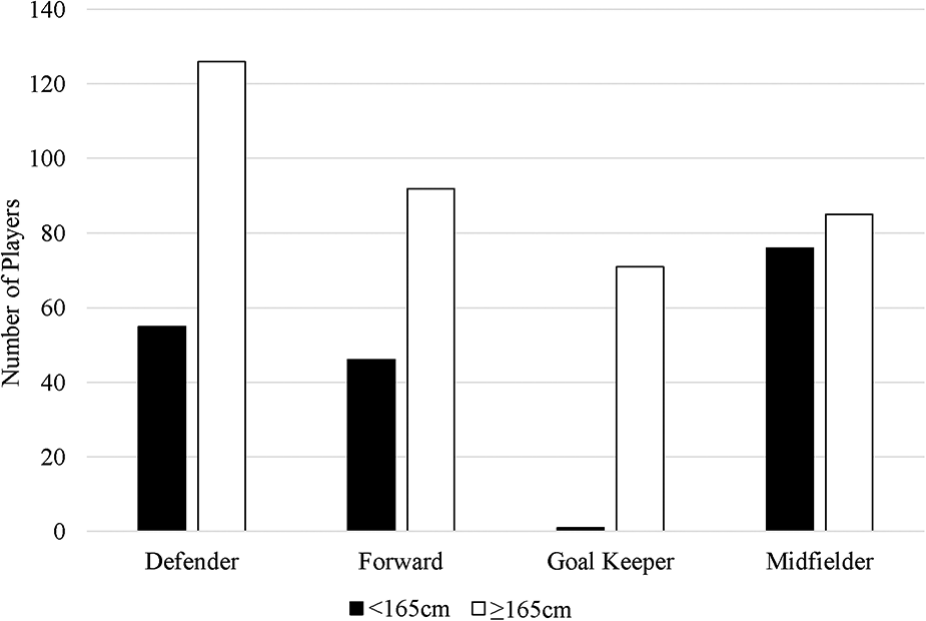
Visual representation of players by position and stature. Black bars indicate number of players <165 cm. White bars indicate number of players ≥165 cm.

Figure 2 gives a visual representation of the number of players in each position. Midfielders had significantly lower stature than defenders [MD = −2.83 (−4.07, −1.58) cm *p *= 0.00], forwards [MD = −2.02 (−3.35, −0.68) cm, *p *= 0.003], and goal keepers [MD = −8.35 (4.66, 8.01) cm, *p *< 0.001], respectively. Goal keepers had significantly greater stature than all other positions; defenders, [MD = 5.53 (3.92, 7.14) cm, *p *< 0.001]; forwards, [MD = 6.34 (4.66, 8.01) cm, *p *< 0.001], midfielders, (refer to previous sentence). No statistically significant differences were found between the two stature groups and playing time among defenders, forwards, midfielders, or goal keepers (*p > 0.05*). ES was small (Table 2).

### Performance

Of the 93 athletes who played ≥ 60 min when entered into a match and were <165 cm tall, 31 (33.33%) were defenders, 18 (19.35%) were forwards, and 44 (47.31%) were midfielders. 20.34% of all goals (24 out of 118), and 28.77% of all assists (21 out of 73) were made by players <165 cm. In comparison of the two stature groups, 93 players <165 cm scored 24 goals and made 21 assists, whereas 195 players ≥165 cm scored 94 goals and made 52 assists. There were no significant differences between players ≥165 cm and players <165 cm in regard to the number of assists among both forwards and defenders (MD [95%CI] ES; forwards, 0.17[−0.21,0.54] 0.29, *p* = 0.375; defenders, 0.03[−0.19,0.24] 0.04, *p* = 0.804). No statistically significant difference was found in the number of goals scored among forwards of the two stature groups (MD [95%CI] ES; 0.41[−1.02,0.20]0.38, *p* = 0.183). Among midfielders in the two stature groups, a significant difference was found in the number of assists and the number of goals made (MD [95%CI] ES; number of assists, −0.44[−0.76,−0.11]0.59, *p* = 0.009; number of goals, −0.35[−0.69,−0.01]0.44, *p* = 0.047), with players ≥165 cm showing a higher number of assists and goals. (Supplementary File 1 shows the performance differences reported above among midfielders in each stature group).

### Forwards

No statistically significant difference was found between stature and any factor of performance, *p* > 0.05.

### Midfielders

Midfielders of greater stature showed significantly higher performance in all variables except distance covered per match played. A statistically significant difference was found with moderate and large effects in number of attempts, attempts on target, attempts off target, attempts on target inside area, attempts on target outside of area, number of corners, shots blocked, and defending blocks (MD [95%CI] ES; number of attempts, 3.14 [1.38, 4.90] 0.80, *p* = 0.001; attempts on target, 1.18 [0.41, 1.94] 0.69, *p* = 0.003; attempts off target, 1.01 [0.19, 1.83] 0.55, *p* = 0.016; attempts on target inside area, 0.83 [0.26, 1.41] 0.65, *p* = 0.005; attempts on target outside area, 0.36 [0.05, 0.67] 0.51, *p* = 0.001; number of corners, 2.04 [0.12, 3.95] 0.48, *p* = 0.037; shots blocked, 0.96 [0.40, 1.51] 0.75, *p* = 0.001; defending blocks, 0.43 [0.32,0.82] 0.48, *p* = 0.035). No statistically significant difference was found in average distance covered per match played (MD [95%CI] ES; average distance covered per match played, 0.29 [−0.52, 1.10] 0.15, *p* = 0.484).

### Defenders

No statistical significance was found in any performance variables for defenders between stature groups, *p* > 0.05.

### Countries

On average, 32.25% of players on the 2019 World Cup team rosters were <165 cm, ranging from China with 4.35% of total players <165 cm, to South Africa with 69.57% of total players <165 cm. Six out of the 24 teams were composed of 50% or more of their starters being <165 cm. Twelve out of the 24 teams had a 35% or greater number of starters that were <165 cm. Supplementary File 2 shows the number and percentage of players <165 cm from each country. On average, athletes from all teams and countries played 190.03 ± 174.09 total minutes, 2.62 ± 1.95 matches, 53.48 ± 36.62 average minutes per match, 43.19 ± 35.75 average minutes in relation to total number of matches team played, and 47.98 ± 39.72 percent of matches. There were no statistically significant differences between countries in average minutes played per match, average minutes played in relation to the total number of matches the team played, or percent playing time, respectively; (*F-*value, *p-*value); average minutes played per match (*F* = 0.34, *p* = 1.00); average minutes played in relation to the total number of matches the team played (*F* = 0.11, *p *= 1.00); percent playing time (*F* = 0.11, *p *= 1.00). Supplementary File 3 shows the breakdown of playing time for players from each country in the 2019 FIFA Women's World Cup.

## Discussion

The primary aim of this study was to determine if a stature of 165 cm (5′5″) is a limitation to the duration of match-playing time on an elite World Cup soccer team, to explore the idea that elite success is in some way related to match-playing time. The secondary aim of this study was to determine if differences in performance exist among elite female soccer players of different stature (≥165 cm and <165 cm) and position in the 2019 FIFA Women's World Cup. Our findings indicate that stature is not a limitation to the duration of match-playing time on an elite World Cup team. Stature did not impact match-playing time at the elite World Cup level in any variables including the odds of starting, minutes played, matches played, average minutes played when entered into a match, average minutes played in relation to the total number of matches the team played in during the World Cup, or percentage of playing time. One-third of players in the 2019 FIFA World Cup were <165 cm. Furthermore, the average height of females ages 20–29 in the world is 162.56 cm (5′4″) (41). In fact, only females in the top 25th percentile are above this height criterion of 165 cm (5′5″) (41). Our data do not justify the criterion value of 165 cm that recruiting companies are using.

Additionally, our findings suggest that stature does not have an impact on performance as minimal differences were observed between stature groups in midfielders, and no differences were observed between stature groups in forwards and defenders. Midfielders of greater stature only exhibited a 0.59 greater number of assists and a 0.44 greater number of goals on average compared to their shorter counterparts. Stature was not an imperative factor in any variable when assessing performance in both forwards and defenders. Although goals scored by players ≥165 cm were more than double that of players <165 cm, the actual difference was minimal when applied practically, with <1.0 goal difference on average per match between players <165 cm and players ≥165 cm (Table 2). Additionally, many players scored zero performance variables, which is reflected in the large confidence intervals found in statistical analyses. The minimal differences found between stature in midfielders may be a result of biomechanics, as longer limbs may attribute to different magnitudes of movement patterns in running, kicking, jumping, and changing direction (cutting ability) (19–21, 23, 42). However, many factors aside from biomechanics have been studied in relation to soccer performance, therefore the contribution of these biomechanical factors alone is not enough to conclude that these elicited the minimal increase found in number of assists and goals among midfielders of greater stature.

A statistically significant difference among midfielders was seen in number of assists, goals scored, attempts, attempts on target, attempts on target inside area, attempts on target outside of area, number of corners, shots blocked, and defending blocks. However, considering players in the 2019 FIFA Women's World Cup ≥165 cm (*n* = 135) were greater in numbers compared to players <165 cm (*n* = 93), this could also explain why midfielders of greater stature exhibited higher performance in all variables. This also addresses the performance of forwards <165 cm, as there were no significant differences in any performance variables regardless of there being more forwards ≥165 cm. In total, there were more attempts made in general by forwards ≥165 cm, however, when compared to the proportion of attempts that were on target and attempts that were off target, the trend was almost identical to that of forwards <165 cm, indicating no difference in performance among players of different stature.

Our findings support previous researchers Lago-Peñas et al. 2011 and Leão et al. 2019 indicating that forwards and midfielders tend to be of smaller, leaner stature and defenders and goal keepers tend to be of greater stature (25, 28). More importantly, our findings support that shorter stature in forwards and midfielders, and being of smaller stature, does not inhibit performance in this position (5, 28, 43). Our study found controversial results from Rebelo et al. 2013 and Buchheit et al. 2010, that have reported taller and heavier anthropometry is critical for success among defenders, with our findings suggesting that stature is not imperative in performance among defenders (5, 43). The aforementioned researchers assessed stature and body mass in youth and adolescent elite male soccer players under the age of 19 and 15 years, whereas our analysis was among world classified elite female soccer players with an average age of 27.14 ± 3.96 years of age, therefore maturation may explain the difference in results (5, 28, 43–45). Biological maturity may have also been cause for the difference in our study compared to others, as mentioned by Malina et al., this accounts for 21% to 50% of variance seen in youth soccer performance (45). While our analysis suggests stature is of minimal influence in performance, it is only one variable. There are many other variables that need to be accounted for when determining if a player will be successful at the elite level including technical match performance, attack factors, and cognitive, perceptual, and motor skills (7, 25, 46, 47). Taking all of this into account, it is clear that there are many factors that influence performance in soccer.

The importance of stature for elite success in terms of match-playing time and performance in an international soccer competition is not clear in scientific research thus far. There is currently no consensus on which variables have the most impact in determining which potential student athletes are selected or receive the most match-playing time once selected (16). Researchers have recently determined the level of crossover that several criterion variables have in the actual playing of soccer, and physical variables such as stature were deemed to have little implication (18). Our findings support previous literature that emphasizes individual skill capabilities rather than physical variables such as stature when identifying talent in potential student athletes, as no difference in match-playing time exists once athletes are selected (15, 16, 48). Our study adds to current soccer literature as no studies have assessed the influence of stature on match-playing time.

The main limitation of this study is the lack of inclusion of other anthropometric variables to consider in addition to height. There are many other variables that need to be accounted for when determining if a player will be successful at the elite level. In addition, the height was captured from the database and it was not directly measured. No body mass was listed on the FIFA website for any players, therefore anthropometric measures of body mass were not able to be accounted for in our analysis. A second limitation of this study is that the analysis mainly included offensive performance variables typically outside the role of defenders. An analysis of more defensive variables may be more representative of performance in defenders. The amount of time each team spent defending was not considered which could have impacted performance variables if teams spent a majority of time during matches defending rather than attacking. Finally, this analysis included players from all teams in the 2019 FIFA Women's World Cup, which included 24 different teams from 24 different countries. Training experience and background may have varied greatly between different teams as players may have learned many different approaches and techniques when performing different skills that could have impacted the performance outcome measures used in this study. This may have influenced the performance of players in their ability of making assists, goals, and other offensive performance variables, regardless of stature. Performance of players of different positions between different countries was not analysed, therefore, training experience and background was not accounted for in this analysis.

These findings make clear that stature does not impact the duration of match-playing time or performance on an elite Women's World Cup soccer team. Recruiting companies suggest that aspiring female soccer players be ≥165 cm, however, 32.3% of elite female soccer players in the 2019 World Cup are below this stature criterion. These guidelines could potentially discourage coaches from selecting players <165 cm and discourage aspiring youth female soccer players from pursuing and advancing in the sport (4). Conveying this information and educating coaches on such scientific evidence will help them better understand that stature does not impact an athlete's performance when playing in a match. Furthermore, deconstructing obsolete stereotypes, such as stature, that are placed on female soccer players is essential in developing the future direction of the sport. Additionally, this will help evolve the sport of soccer ensuring that all female athletes are given a chance to acquire and prove their skill, before being excluded merely based on stature.

## Data Availability

The original contributions presented in the study are included in the article/**Supplementary Material**, further inquiries can be directed to the corresponding author/s.
