# Supplementary material for: Deconstructing stereotypes: Stature, match-playing time, and performance in elite Women's World Cup soccer
Source: Front Sports Act Living. 2022 Dec 14;4:1067190. doi: 10.3389/fspor.2022.1067190 (PMC9795175; doi:10.3389/fspor.2022.1067190)
Supplement: Supplementary file 1 [file Presentation1.zip › FIFAWC_Figures_10112022.docx]

**Figures**

Figure 1. Overall breakdown of the statures of 552 players representing 24 countries in the 2019 FIFA Women’s World Cup games.

Figure 2. Visual representation of players by position and stature. Black bars indicate number of players <165 cm. White bars indicate number of players ≥165 cm.
