# Supplementary material for: Deconstructing stereotypes: Stature, match-playing time, and performance in elite Women's World Cup soccer
Source: Front Sports Act Living. 2022 Dec 14;4:1067190. doi: 10.3389/fspor.2022.1067190 (PMC9795175; doi:10.3389/fspor.2022.1067190)
Supplement: Supplementary file 1 [file Presentation1.zip › Supplemental_Files_10.11.2022.docx]

**Supplemental Files**

Supplementary File 1. Comparison of Average Number of Different Performance Variables Among Players <165cm and Players ≥165cm in all Positions.

|  | **Forwards <165cm** | **Forwards ≥165cm** | **Midfielders <165cm** | **Midfielders ≥165cm** | **Defenders <165cm** | **Defenders ≥165cm** |
| --- | --- | --- | --- | --- | --- | --- |
| **Goals** | 0.78 ± 0.73 | 1.19 ± 1.73 | 0.23 ± 0.68 | 0.57 ± 0.87* | 0.00 ± 0.00 | 0.18 ± 0.55* |
| **Assists** | 0.50 ± 0.51 | 0.33 ± 0.72 | 0.14 ± 0.51 | 0.57 ± 0.93* | 0.19 ± 0.60 | 0.17 ± 0.47 |
| **Attempts** | 7.39 ± 4.16 | 7.96 ± 6.55 | 2.91 ± 2.94 | 6.05 ± 4.81* | 1.36 ± 1.71 | 1.61 ± 1.97 |
| **Attempts on Target** | 2.56 ± 1.98 | 3.29 ± 3.57 | 0.77 ± 1.26 | 1.95 ± 2.11* | 0.33 ± 0.69 | 0.53 ± 1.04 |
| **Attempts inside the area** | 4.47 ± 3.17 | 5.19 ± 5.08 | 0.95 ± 1.60 | 2.73 ± 3.23* | 0.36 ± 0.70 | 0.93 ± 1.26* |
| **Attempts outside the area** | 2.82 ± 1.67 | 2.33 ± 2.44 | 1.66 ± 1.72 | 2.93 ± 2.43* | 0.85 ± 1.03 | 0.62 ± 0.99 |
| **Attempts on target inside the area** | 1.88 ± 1.54 | 2.69 ± 3.06 | 0.34 ± 0.89 | 1.18 ± 1.62* | 0.15 ± 0.44 | 0.38 ± 0.82 |
| **Attempts on target outside the area** | 0.71 ± 0.59 | 0.52 ± 0.92 | 0.34 ± 0.57 | 0.70 ± 0.82* | 0.15 ± 0.36 | 0.18 ± 0.51 |
| **Corners** | 1.94 ± 3.30 | 2.27 ± 5.35 | 1.14 ± 2.45 | 3.18 ± 5.57* | 0.82 ± 3.66 | 0.75 ± 2.86 |
| **Shots blocked** | 1.78 ± 1.63 | 1.56 ± 1.65 | 0.80 ± 1.19 | 1.75 ± 1.35 | 0.36 ± 0.82 | 0.26 ± 0.57 |
| **Defensive blocks** | 0.06 ± 0.24 | 0.25 ± 0.57 | 0.52 ± 0.70 | 0.95 ± 1.06* | 1.42 ± 1.62 | 1.79 ± 1.97 |
| **Distance per match played** | 8.12 ± 1.99 | 8.81 ± 1.57 | 9.02 ± 2.19 | 9.31 ± 1.42 | 9.36 ± 0.92 | 9.24 ± 0.91 |

*Indicates statistically significant differences (p<0.05) between stature groups

Averages were calculated by dividing the number of each performance variables (e.g. number of goals scored by forwards <165cm) by the total number of players in that position and stature group (e.g. number of forwards <165cm).

Supplemental File 2. List of teams by country and number and percentage of starters <165 cm.

| **Team/Country** | **# of starters** | **# of starters**  **<165 cm** | **% of starters**  **<165 cm** |
| --- | --- | --- | --- |
| Argentina | 12 | 5 | 41.67 |
| Australia | 10 | 2 | 20 |
| Brazil | 12 | 6 | 50 |
| Cameroon | 14 | 9 | 75 |
| Canada | 12 | 5 | 41.67 |
| Chile | 11 | 5 | 45.45 |
| China | 11 | 0 | 0 |
| England | 14 | 3 | 21.43 |
| France | 11 | 4 | 36.36 |
| Germany | 12 | 1 | 8.33 |
| Italy | 11 | 4 | 36.36 |
| Jamaica | 15 | 2 | 13.33 |
| Japan | 12 | 8 | 66.67 |
| Korea | 13 | 5 | 38.46 |
| Netherlands | 12 | 1 | 8.30 |
| New Zealand | 10 | 5 | 50 |
| Nigeria | 13 | 3 | 23.08 |
| Norway | 11 | 2 | 18.18 |
| Scotland | 14 | 4 | 28.57 |
| South Africa | 14 | 10 | 71.43 |
| Spain | 12 | 2 | 16.67 |
| Sweden | 10 | 0 | 0 |
| Thailand | 11 | 6 | 54.55 |
| USA | 13 | 3 | 23.08 |
| **AVERAGE**  **(M±SD)** | 12.08 ± 1.41 | 3.96 ± 2.63 | 33.08 ± 21.53 |

Mean ± Standard Deviation (M ± SD)

Players were considered starters if they played ≥60 minutes when entered into a match.

Supplementary File 3. Mean and standard deviation (M ± SD) playing time measures of players from all countries that played in the 2019 FIFA Women’s World Cup.

| **Team/Country** | **Total minutes played** | **Number of matches played** | **Average minutes played per match** | **Average minutes in relation to total matches team played** | **Percent playing time** |
| --- | --- | --- | --- | --- | --- |
| Argentina | 129.13 ± 114.20 | 1.82 ± 1.30 | 50.06 ± 38.45 | 43.04 ± 38.07 | 47.83 ± 42.30 |
| Australia | 185.83 ± 161.40 | 2.43 ± 1.62 | 52.76 ± 40.25 | 46.46 ± 40.35 | 51.62 ± 44.83 |
| Brazil | 186.52 ± 158.45 | 2.48 ± 1.62 | 53.75 ± 39.45 | 46.63 ± 39.61 | 51.81 ± 44.01 |
| Cameroon | 172.17 ± 140.04 | 2.39 ± 1.44 | 57.47 ± 34.85 | 43.04 ± 35.01 | 47.83 ± 38.90 |
| Canada | 172.17 ± 163.87 | 2.35 ± 1.80 | 47.93 ± 41.34 | 43.04 ± 40.97 | 47.83 ± 45.52 |
| Chile | 129.13 ± 114.27 | 1.74 ± 1.21 | 50.88 ± 38.93 | 43.04 ± 38.09 | 47.83 ± 42.32 |
| China | 172.17 ± 151.54 | 2.43 ± 1.75 | 48.99 ± 37.01 | 43.04 ± 37.89 | 47.83 ± 42.09 |
| England | 301.17 ± 200.35 | 4.26 ± 1.98 | 62.20 ± 31.36 | 43.02 ± 28.62 | 47.81 ± 31.80 |
| France | 229.57 ± 203.29 | 3.00 ± 2.15 | 50.71 ± 41.36 | 32.80 ± 29.04 | 36.44 ± 32.27 |
| Germany | 215.22 ± 172.28 | 3.04 ± 1.94 | 55.50 ± 31.66 | 43.04 ± 34.46 | 47.83 ± 38.28 |
| Italy | 215.22 ± 187.81 | 3.04 ± 2.20 | 46.87 ± 36.02 | 43.04 ± 37.56 | 47.83 ± 41.74 |
| Jamaica | 129.13 ± 103.20 | 1.83 ± 1.03 | 60.93 ± 33.11 | 43.04 ± 30.18 | 47.83 ± 33.53 |
| Japan | 172.22 ± 151.09 | 2.35 ± 1.70 | 51.09 ± 38.46 | 43.05 ± 37.77 | 47.84 ± 41.97 |
| Korea | 129.13 ± 103.20 | 1.83 ± 1.03 | 55.58 ± 36.32 | 43.04 ± 34.40 | 47.83 ± 38.22 |
| Netherlands | 315.65 ± 281.22 | 4.17 ± 3.20 | 49.34 ± 41.06 | 45.09 ± 40.17 | 50.10 ± 44.64 |
| New Zealand | 129.13 ± 118.20 | 1.83 ± 1.30 | 47.41 ± 38.47 | 43.04 ± 39.40 | 47.83 ± 43.78 |
| Nigeria | 171.48 ± 139.88 | 2.43 ± 1.50 | 56.63 ± 35.43 | 42.87 ± 34.97 | 47.63 ± 38.86 |
| Norway | 229.57 ± 220.84 | 3.04 ± 2.23 | 47.82 ± 43.16 | 45.91 ± 44.17 | 51.01 ± 49.08 |
| Scotland | 129.13 ± 111.17 | 1.78 ± 1.17 | 55.97 ± 37.99 | 43.04 ± 37.06 | 47.83 ± 41.17 |
| South Africa | 128.74 ± 98.86 | 1.83 ± 1.11 | 57.93 ± 35.48 | 42.91 ± 32.95 | 47.68 ± 36.61 |
| Spain | 172.17 ± 140.92 | 2.43 ± 1.59 | 53.53 ± 34.73 | 43.04 ± 35.23 | 47.83 ± 39.14 |
| Sweden | 315.65 ± 250.62 | 4.30 ± 2.49 | 56.86 ± 33.78 | 45.09 ± 35.80 | 50.10 ± 39.78 |
| Thailand | 129.13 ± 108.07 | 1.83 ± 1.15 | 52.67 ± 37.97 | 43.04 ± 36.02 | 47.83 ± 40.02 |
| USA | 301.30 ± 223.17 | 4.26 ± 2.61 | 60.50 ± 31.21 | 43.04 ± 31.88 | 47.83 ± 39.72 |
| **Total Average**  **(M±SD)** | 190.03 ± 174.09 | 2.62 ± 1.95 | 53.48 ± 36.62 | 43.19 ± 35.75 | 47.98 ± 39.72 |
