# Supplementary material for: Deconstructing stereotypes: Stature, match-playing time, and performance in elite Women's World Cup soccer
Source: Front Sports Act Living. 2022 Dec 14;4:1067190. doi: 10.3389/fspor.2022.1067190 (PMC9795175; doi:10.3389/fspor.2022.1067190)
Supplement: Supplementary file 2 [file Presentation2.zip › Supplementary Files/Supplementary File 1.docx]

Supplementary File 1. Comparison of Average Number of Different Performance Variables Among Players <165cm and Players ≥165cm in all Positions.

|  | **Forwards <165cm** | **Forwards ≥165cm** | **Midfielders <165cm** | **Midfielders ≥165cm** | **Defenders <165cm** | **Defenders ≥165cm** |
| --- | --- | --- | --- | --- | --- | --- |
| **Goals** | 0.78 ± 0.73 | 1.19 ± 1.73 | 0.23 ± 0.68 | 0.57 ± 0.87* | 0.00 ± 0.00 | 0.18 ± 0.55* |
| **Assists** | 0.50 ± 0.51 | 0.33 ± 0.72 | 0.14 ± 0.51 | 0.57 ± 0.93* | 0.19 ± 0.60 | 0.17 ± 0.47 |
| **Attempts** | 7.39 ± 4.16 | 7.96 ± 6.55 | 2.91 ± 2.94 | 6.05 ± 4.81* | 1.36 ± 1.71 | 1.61 ± 1.97 |
| **Attempts on Target** | 2.56 ± 1.98 | 3.29 ± 3.57 | 0.77 ± 1.26 | 1.95 ± 2.11* | 0.33 ± 0.69 | 0.53 ± 1.04 |
| **Attempts inside the area** | 4.47 ± 3.17 | 5.19 ± 5.08 | 0.95 ± 1.60 | 2.73 ± 3.23* | 0.36 ± 0.70 | 0.93 ± 1.26* |
| **Attempts outside the area** | 2.82 ± 1.67 | 2.33 ± 2.44 | 1.66 ± 1.72 | 2.93 ± 2.43* | 0.85 ± 1.03 | 0.62 ± 0.99 |
| **Attempts on target inside the area** | 1.88 ± 1.54 | 2.69 ± 3.06 | 0.34 ± 0.89 | 1.18 ± 1.62* | 0.15 ± 0.44 | 0.38 ± 0.82 |
| **Attempts on target outside the area** | 0.71 ± 0.59 | 0.52 ± 0.92 | 0.34 ± 0.57 | 0.70 ± 0.82* | 0.15 ± 0.36 | 0.18 ± 0.51 |
| **Corners** | 1.94 ± 3.30 | 2.27 ± 5.35 | 1.14 ± 2.45 | 3.18 ± 5.57* | 0.82 ± 3.66 | 0.75 ± 2.86 |
| **Shots blocked** | 1.78 ± 1.63 | 1.56 ± 1.65 | 0.80 ± 1.19 | 1.75 ± 1.35 | 0.36 ± 0.82 | 0.26 ± 0.57 |
| **Defensive blocks** | 0.06 ± 0.24 | 0.25 ± 0.57 | 0.52 ± 0.70 | 0.95 ± 1.06* | 1.42 ± 1.62 | 1.79 ± 1.97 |
| **Distance per match played** | 8.12 ± 1.99 | 8.81 ± 1.57 | 9.02 ± 2.19 | 9.31 ± 1.42 | 9.36 ± 0.92 | 9.24 ± 0.91 |

*Indicates statistically significant differences (p<0.05) between stature groups

Averages were calculated by dividing the number of each performance variables (e.g. number of goals scored by forwards <165cm) by the total number of players in that position and stature group (e.g. number of forwards <165cm).
