# Supplementary material for: Deconstructing stereotypes: Stature, match-playing time, and performance in elite Women's World Cup soccer
Source: Front Sports Act Living. 2022 Dec 14;4:1067190. doi: 10.3389/fspor.2022.1067190 (PMC9795175; doi:10.3389/fspor.2022.1067190)
Supplement: Supplementary file 2 [file Presentation2.zip › Supplementary Files/Supplementary File 3.docx]

Supplementary File 3. Mean and standard deviation (M ± SD) playing time measures of players from all countries that played in the 2019 FIFA Women’s World Cup.

| **Team/Country** | **Total minutes played** | **Number of matches played** | **Average minutes played per match** | **Average minutes in relation to total matches team played** | **Percent playing time** |
| --- | --- | --- | --- | --- | --- |
| Argentina | 129.13 ± 114.20 | 1.82 ± 1.30 | 50.06 ± 38.45 | 43.04 ± 38.07 | 47.83 ± 42.30 |
| Australia | 185.83 ± 161.40 | 2.43 ± 1.62 | 52.76 ± 40.25 | 46.46 ± 40.35 | 51.62 ± 44.83 |
| Brazil | 186.52 ± 158.45 | 2.48 ± 1.62 | 53.75 ± 39.45 | 46.63 ± 39.61 | 51.81 ± 44.01 |
| Cameroon | 172.17 ± 140.04 | 2.39 ± 1.44 | 57.47 ± 34.85 | 43.04 ± 35.01 | 47.83 ± 38.90 |
| Canada | 172.17 ± 163.87 | 2.35 ± 1.80 | 47.93 ± 41.34 | 43.04 ± 40.97 | 47.83 ± 45.52 |
| Chile | 129.13 ± 114.27 | 1.74 ± 1.21 | 50.88 ± 38.93 | 43.04 ± 38.09 | 47.83 ± 42.32 |
| China | 172.17 ± 151.54 | 2.43 ± 1.75 | 48.99 ± 37.01 | 43.04 ± 37.89 | 47.83 ± 42.09 |
| England | 301.17 ± 200.35 | 4.26 ± 1.98 | 62.20 ± 31.36 | 43.02 ± 28.62 | 47.81 ± 31.80 |
| France | 229.57 ± 203.29 | 3.00 ± 2.15 | 50.71 ± 41.36 | 32.80 ± 29.04 | 36.44 ± 32.27 |
| Germany | 215.22 ± 172.28 | 3.04 ± 1.94 | 55.50 ± 31.66 | 43.04 ± 34.46 | 47.83 ± 38.28 |
| Italy | 215.22 ± 187.81 | 3.04 ± 2.20 | 46.87 ± 36.02 | 43.04 ± 37.56 | 47.83 ± 41.74 |
| Jamaica | 129.13 ± 103.20 | 1.83 ± 1.03 | 60.93 ± 33.11 | 43.04 ± 30.18 | 47.83 ± 33.53 |
| Japan | 172.22 ± 151.09 | 2.35 ± 1.70 | 51.09 ± 38.46 | 43.05 ± 37.77 | 47.84 ± 41.97 |
| Korea | 129.13 ± 103.20 | 1.83 ± 1.03 | 55.58 ± 36.32 | 43.04 ± 34.40 | 47.83 ± 38.22 |
| Netherlands | 315.65 ± 281.22 | 4.17 ± 3.20 | 49.34 ± 41.06 | 45.09 ± 40.17 | 50.10 ± 44.64 |
| New Zealand | 129.13 ± 118.20 | 1.83 ± 1.30 | 47.41 ± 38.47 | 43.04 ± 39.40 | 47.83 ± 43.78 |
| Nigeria | 171.48 ± 139.88 | 2.43 ± 1.50 | 56.63 ± 35.43 | 42.87 ± 34.97 | 47.63 ± 38.86 |
| Norway | 229.57 ± 220.84 | 3.04 ± 2.23 | 47.82 ± 43.16 | 45.91 ± 44.17 | 51.01 ± 49.08 |
| Scotland | 129.13 ± 111.17 | 1.78 ± 1.17 | 55.97 ± 37.99 | 43.04 ± 37.06 | 47.83 ± 41.17 |
| South Africa | 128.74 ± 98.86 | 1.83 ± 1.11 | 57.93 ± 35.48 | 42.91 ± 32.95 | 47.68 ± 36.61 |
| Spain | 172.17 ± 140.92 | 2.43 ± 1.59 | 53.53 ± 34.73 | 43.04 ± 35.23 | 47.83 ± 39.14 |
| Sweden | 315.65 ± 250.62 | 4.30 ± 2.49 | 56.86 ± 33.78 | 45.09 ± 35.80 | 50.10 ± 39.78 |
| Thailand | 129.13 ± 108.07 | 1.83 ± 1.15 | 52.67 ± 37.97 | 43.04 ± 36.02 | 47.83 ± 40.02 |
| USA | 301.30 ± 223.17 | 4.26 ± 2.61 | 60.50 ± 31.21 | 43.04 ± 31.88 | 47.83 ± 39.72 |
| **Total Average**  **(M±SD)** | 190.03 ± 174.09 | 2.62 ± 1.95 | 53.48 ± 36.62 | 43.19 ± 35.75 | 47.98 ± 39.72 |
