# Supplementary material for: Deconstructing stereotypes: Stature, match-playing time, and performance in elite Women's World Cup soccer
Source: Front Sports Act Living. 2022 Dec 14;4:1067190. doi: 10.3389/fspor.2022.1067190 (PMC9795175; doi:10.3389/fspor.2022.1067190)
Supplement: Supplementary file 2 [file Presentation2.zip › Supplementary Files/Supplemental File 2.docx]

Supplemental File 2. List of teams by country and number and percentage of starters <165 cm.

| **Team/Country** | **# of starters** | **# of starters**  **<165 cm** | **% of starters**  **<165 cm** |
| --- | --- | --- | --- |
| Argentina | 12 | 5 | 41.67 |
| Australia | 10 | 2 | 20 |
| Brazil | 12 | 6 | 50 |
| Cameroon | 14 | 9 | 75 |
| Canada | 12 | 5 | 41.67 |
| Chile | 11 | 5 | 45.45 |
| China | 11 | 0 | 0 |
| England | 14 | 3 | 21.43 |
| France | 11 | 4 | 36.36 |
| Germany | 12 | 1 | 8.33 |
| Italy | 11 | 4 | 36.36 |
| Jamaica | 15 | 2 | 13.33 |
| Japan | 12 | 8 | 66.67 |
| Korea | 13 | 5 | 38.46 |
| Netherlands | 12 | 1 | 8.30 |
| New Zealand | 10 | 5 | 50 |
| Nigeria | 13 | 3 | 23.08 |
| Norway | 11 | 2 | 18.18 |
| Scotland | 14 | 4 | 28.57 |
| South Africa | 14 | 10 | 71.43 |
| Spain | 12 | 2 | 16.67 |
| Sweden | 10 | 0 | 0 |
| Thailand | 11 | 6 | 54.55 |
| USA | 13 | 3 | 23.08 |
| **AVERAGE**  **(M±SD)** | 12.08 ± 1.41 | 3.96 ± 2.63 | 33.08 ± 21.53 |

Mean ± Standard Deviation (M ± SD)

Players were considered starters if they played ≥60 minutes when entered into a match.
